# Evaluating prognostic value of the prognostic nutritional index, CRP-to-albumin ratio and lymphocyte-to-CRP ratio in patients with idiopathic pulmonary fibrosis

**DOI:** 10.1080/07853890.2026.2689565

**Published:** 2026-06-26

**Authors:** Qing Zhang, Xiaoli Ouyang, Yuexin Tan, Yijin Qian, Min Song, Hong Peng

**Affiliations:** ^a^ Department of Pulmonary and Critical Care Medicine, the Second Xiangya Hospital, Central South University, Changsha, China; ^b^ Research Unit of Respiratory Disease, Central South University, Changsha, China; ^c^ Clinical Medical Research Center for Pulmonary and Critical Care Medicine in Hunan Province, Changsha, China; ^d^ Diagnosis and Treatment Center of Respiratory Disease in Hunan Province, Changsha, China

**Keywords:** Idiopathic pulmonary fibrosis, prognosis, prognostic nutritional index, C-reactive protein to albumin ratio, lymphocyte to C-reactive protein ratio

## Abstract

**Background:**

The clinical outcome of patients with idiopathic pulmonary fibrosis (IPF) exhibit significant variability. Inflammation, immune response, and nutritional status are critical factors influencing the progression of IPF. Consequently, the identification of biomarkers that integrate these three elements is essential for evaluating prognosis and optimizing treatment strategies in IPF patients.

**Methods:**

We analyzed and compared baseline data from 240 IPF patients at the Second Xiangya Hospital of Central South University, covering the period from January 2011 to December 2020. Correlation analyses were conducted to assess the relationship between the prognostic nutritional index (PNI), C-reactive protein (CRP) to albumin ratio (CAR) and lymphocyte to CRP ratio (LCR) with pulmonary function, age, and body mass index (BMI). The predictive accuracy of PNI, CAR and LCR was assessed using Kaplan-Meier survival curves, receiver operating characteristic (ROC) curves, and calibration curves. Potential risk factors were identified through Cox regression analysis.

**Results:**

Non-survivors and IPF-related deaths exhibited lower PNI and LCR and higher CAR compared to survivors. PNI demonstrated the strongest correlation with age and BMI, while LCR was most strongly correlated with pulmonary function. In terms of predictive ability, PNI outperformed CAR and LCR for both overall and IPF-related mortality. Univariate Cox regression analyses indicated that PNI, CAR, and LCR effectively discriminated between overall survival (OS) and IPF-related mortality. However, in multivariate analyses, PNI remained the sole independent predictor of OS and IPF-related mortality.

**Conclusion:**

PNI serves as a robust prognostic indicator for OS and IPF-related mortality in patients with IPF, demonstrating superior predictive capability compared to CAR and LCR.

## Introduction

Idiopathic pulmonary fibrosis (IPF) is a chronic, progressive lung disease of unknown etiology, characterized by alveolar damage, fibroblast proliferation, excessive extracellular matrix deposition, and irreversible remodeling of lung tissue [1]. Despite the introduction of antifibrotic agents such as pirfenidone and nintedanib, their efficacy remains limited, primarily serving to slow disease progression rather than halt it. The prognosis for patients with IPF is generally poor, with median survival estimated at 2–3 years. The global prevalence of IPF is on the rise, contributing significantly to the socioeconomic burden associated with respiratory diseases [2].

Chronic inflammation is the main feature of IPF [3]. C-reactive protein (CRP), an acute-phase reactant primarily synthesized by the liver, serves as a critical marker of systemic inflammation [4]. Elevated circulating CRP levels are commonly observed in IPF and have been associated with reduced survival, particularly during acute exacerbations of the disease [5]. Inflammation involves a highly coordinated network of immune-related cells driven by innate and adaptive immune responses in which lymphocytes play a pivotal role. Additionally, serum albumin (ALB) levels provide insight into nutritional status and inflammatory response [6], with lower levels correlating with increased mortality across various diseases. Zisman et al. reported that in patients with idiopathic interstitial pneumonitis awaiting lung transplantation in the United States, decreased serum ALB levels independently correlated with higher mortality [7]. Consequently, inflammation, immune response, and nutritional status are critical factors influencing the progression and prognosis of IPF.

Previous research on IPF biomarkers has mostly focused on single inflammatory or nutritional indicators, without in-depth exploration of the value of composite indices that integrate nutrition, inflammation, and immunity [8,9]. The prognostic nutritional index (PNI) integrates serum ALB levels and peripheral blood lymphocyte counts [10], has been applied in both non-small cell lung cancer and chronic obstructive pulmonary disease (COPD) [11,12]^.^ The CRP to ALB ratio (CAR) has emerged as a prognostic marker for COPD, non-small-cell lung cancer, and severe obstructive sleep apnea [13–15]. Similarly, the lymphocyte-to-CRP ratio (LCR) has been identified as a prognostic indicator across various malignancies and has been adapted for use in other diseases characterized by immune dysregulation and inflammation [16]. As composite indices, PNI, CAR, and LCR can reflect the interrelated pathophysiological processes in IPF (inflammation, malnutrition, and immune dysregulation) – these processes are known to drive disease progression, but there is still a lack of combined research on the three. Furthermore, existing studies mostly focus on single indicators or a limited number of indices, and no study has directly compared these three indices to identify the most robust prognostic indicator in IPF, especially lacking exploration of their interaction with known factors such as pulmonary function and age.

The aim of this retrospective study was to compare the prognostic value of PNI, CAR, and LCR in IPF patients, with a focus on identifying the most robust marker. And we speculate that PNI will exhibit superior prognostic performance compared to CAR and LCR in IPF patients, given its comprehensive reflection of systemic pathophysiological status closely linked to IPF progression.

## Methods

### Study design and participants

This retrospective study analyzed the medical records of 317 patients diagnosed with IPF who were admitted to the Second Xiangya Hospital of Central South University between January 2011 and December 2020. The inclusion criteria for IPF were: (1) Age ≥ 18 years at disease onset; (2) Diagnosis based on the criteria for IPF [17], which include exclusion of other known causes of interstitial lung disease (ILD), the presence of the high-resolution computed tomography (HRCT) pattern of usual interstitial pneumonia (UIP), and specific combinations of HRCT patterns and histopathology patterns in patients who underwent lung tissue sampling; (3) Data on lymphocyte count, CRP and ALB were available within 24 h of admission. Exclusion criteria were: (1) Incomplete data; and (2) Concurrent malignancies.

### Data collection

Clinical data were collected from patients with IPF, including age, gender, body mass index (BMI), smoking status, comorbidities, laboratory findings (including ALB, CRP, neutrophil count, lymphocyte count and platelet count), pulmonary function (including percent of predicted values for forced vital capacity (FVC), percent of predicted values for forceful expiratory volume in 1 s (FEV 1), FEV 1/FVC and diffusion capacity for carbon monoxide (DLCO) as a percentage of predicted value) and treatment modalities. Clinical parameters: age, gender, BMI, smoking status, comorbidities and treatment modalities were extracted from electronic medical records; neutrophil count, lymphocyte count, ALB and CRP were measured by the Clinical Laboratory of the Second Xiangya Hospital of Central South University using standard detection methods (detection instruments: BECKMAN COULTER IMAGE 800 and ABBOTT ARCHITECT C8000, USA) after venous blood collection from fasting subjects. Pulmonary function tests: percent of predicted FVC, percent of predicted FEV 1, FEV 1/FVC and percent of predicted DLCO were measured using the Jaeger MasterScreen pulmonary function system, with each patient completing at least 3 valid tests.

### Definition

IPF-related mortality was determined by reviewing medical records, death certificates, and follow-up records. It specifically refers to deaths directly attributed to IPF progression (e.g. respiratory failure due to worsening interstitial fibrosis, acute exacerbation of IPF), excluding deaths from other causes (e.g. cardiovascular events, infections unrelated to IPF). The follow-up period was defined as the time from admission to death, lung transplantation, or the latest follow-up data available (30 May 2024). Follow-up data were collected annually through inpatient/outpatient visits or telephone contact. Absolute lymphocyte counts, CRP, and ALB levels were obtained *via* routine laboratory blood tests. The following formulas were employed to calculate the prognostic indicators: PNI = ALB (g/L) + 5 × absolute lymphocyte count (× 10^9 cells/L), CAR = CRP (mg/L)/ALB (g/L), LCR = absolute lymphocyte count (× 10^9 cells/L)/CRP (mg/L).

### Statistical analysis

Continuous variables with a normal distribution were expressed as mean ± standard deviation (SD), and intergroup differences were assessed using Student’s *t*-test. Non-normally distributed variables were presented as median (interquartile range, IQR) and compared using the Mann-Whitney U test. Categorical variables were reported as *n* (%) and analyzed using the Pearson chi-square test or Fisher’s exact test. Correlations among variables were evaluated using Pearson or Spearman correlation coefficients. Optimal cut-off values for potential biomarkers were determined through receiver operating characteristic (ROC) analysis, and predictive abilities were assessed *via* calibration curves. Kaplan-Meier survival curves and log-rank tests were utilized to compare cumulative survival rates between groups. Cox regression analysis was performed to evaluate the independent effects of PNI, CAR, and LCR in both unadjusted and adjusted models. The significance level of all tests was 0.05, and all data were analyzed using SPSS version 27.0, GraphPad Prism version 9.0 and R software version 4.0.2.

## Result

### Baseline characteristics of survivors and non-survivors

**Figure 1.**
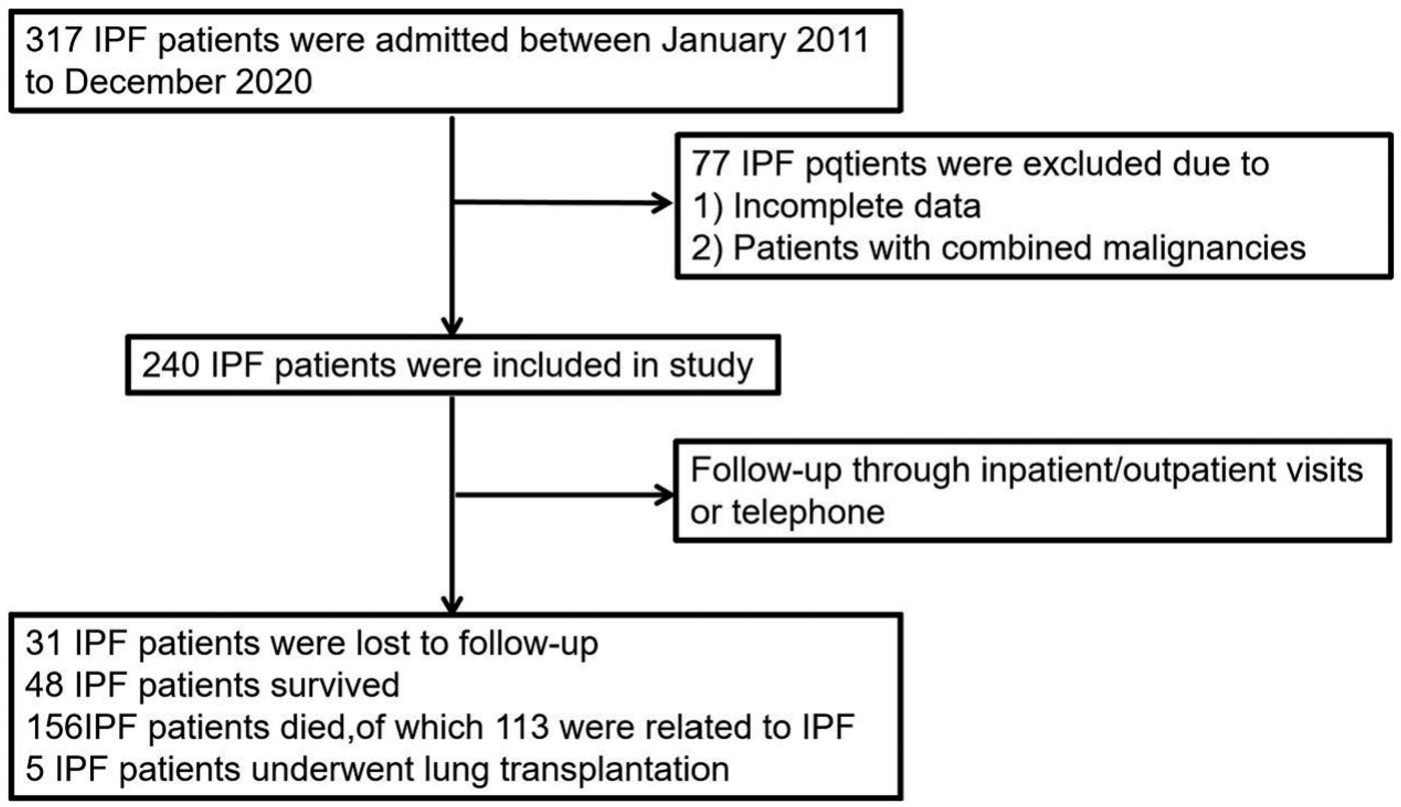
Flow diagram of patients’ selection.

A total of 77 patients were excluded based on these criteria, resulting in 240 patients included in the final analysis. Among these, 31 patients were lost to follow-up, 48 patients survived, 5 patients underwent lung transplantation, and 156 patients died, with 113 deaths related to IPF. Non-survivors include the total number of patients who died or underwent lung transplantation by the end of the follow-up time (Figure 1). The baseline characteristics of the 209 patients diagnosed with IPF are summarised in Table 1, which includes data from 48 survivors and 161 non-survivors. The median age of the cohort was 67 years, with 74.2% of participants being male. Significant differences were observed in PNI, CAR, and LCR values between survivors and 161 non-survivors (*p* < 0.001). Survivors exhibited longer hospital stays, higher BMI, diastolic blood pressure (SBP), ALB and lymphocyte number, along with improved lung function as measured by FVC% of predicted and DLCO% of predicted. In contrast, survivors had lower CRP levels compared to non-survivors. Furthermore, significant differences were noted in the Gender-Age-Physiology (GAP) stage, the presence of comorbid cardiovascular disease (CVD), and the utilization of CVD treatments and anti-acid therapies between survivors and non-survivors (*p* < 0.05).

**Table 1.** Baseline characteristics of patients with survivors and non-survivors.

Variables	Total (*n* = 209)	Survivors (*n* = 48)	Non-survivors (*n* = 161)	*p*
Demographics				
Age	67.0 (59.0–74.0)	57.5 (50.0–65.8)	68.0 (62.5–75.0)	<0.001
Gender				0.548
Male	155 (74.2%)	34 (70.8%)	121 (75.2%)	
Female	54 (25.8%)	14 (29.2%)	40 (24.8%)	
Smoke status				0.841
Current smoking	12 (5.7%)	2 (4.2%)	10 (6.2%)	
Former smoking	126 (60.3%)	30 (62.5%)	96 (59.6%)	
Never smoke	71 (34%)	16 (33.3%)	55 (34.2%)	
BMI, kg/m^2^	23.64 ± 3.42	25.54 ± 3.12	23.07 ± 3.31	<0.001
Comorbidity				
CVD, *n* (%)				<0.001
No	132 (63.2%)	40 (83.3%)	92 (57.1%)	
Yes	77 (36.8%)	8 (16.7%)	69 (42.9%)	
Diabetes, *n* (%)				0.155
No	173 (82.8%)	43 (89.6%)	130 (80.7%)	
Yes	36 (17.2%)	5 (10.4%)	31 (19.3%)	
Pulmonary emphysema, *n* (%)				0.375
No	179 (85.6%)	43 (89.6%)	136 (84.5%)	
Yes	30 (14.4%)	5 (10.4%)	25 (15.5%)	
COPD, *n* (%)				0.547
No	194 (92.8%)	46 (95.8%)	148 (91.9%)	
Yes	15 (7.2%)	2 (4.2%)	13 (8.1%)	
Tuberculosis, *n* (%)				0.123
No	191 (91.4%)	47 (97.9%)	144 (89.4%)	
Yes	18 (8.6%)	1 (2.1%)	17 (10.6%)	
Lung infection				0.083
No	164 (78.5%)	42 (87.5%)	122 (75.8%)	
Yes	45 (21.5%)	6 (12.5%)	39 (24.2%)	
Respiratory failure				0.006
No	286 (89%)	48 (100.0%)	138 (85.7%)	
Yes	23 (11%)	0 (0.0%)	23 (14.3%)	
Laboratory results				
Hemoglobin (g/L)	131.4 ± 17.2	138.3 ± 16.3	129.4 ± 16.9	<0.001
Lymphocyte (×109 /L)	1.80 ± 0.63	1.93 ± 0.54	1.72 ± 0.65	0.043
Neutrophil (×109 /L)	4.55 (3.51–6.34)	4.25 (3.65–5.52)	4.74 (3.47–6.50)	0.153
Platelet (×109/L)	203.0 (162.0–245.0)	211.0 (172.3–240.0)	202.0 (157.5–246.5)	0.365
ALB (g/L)	36.10 (33.05–38.35)	38.20 (35.83–39.78)	35.50 (32.35–37.40)	<0.001
CRP (mg/L)	5.320 (2.895–12.600)	3.235 (2.235–5.393)	6.500 (3.275–15.150)	<0.001
Laboratory results-derived indexes				
PNI	44.800 (40.875–47.900)	47.525 (44.850–49.7)	44.100 (39.858–46.625)	<0.001
CAR	0.147 (0.075–0.363)	0.089 (0.059–0.146)	0.185 (0.090–0.450)	<0.001
LCR	0.323 (0.127–0.614)	0.574 (0.330–0.870)	0.246 (0.107–0.540)	<0.001
Pulmonary function tests				
FVC% of predicted	77.7 ± 19.8	89.1 ± 18.4	77.2 ± 21.4	<0.001
FEV1% of predicted	82.0 ± 19.7	89.9 ± 19.8	80.5 ± 19.2	0.003
DLCO% of predicted	53.9 ± 17.8	64.1 ± 18.5	51.6 ± 16.3	<0.001
FEV1/FVC	0.836 (0.780–0.890)	0.819 (0.771–0.855)	0.839 (0.783–0.907)	0.077
GAP index				<0.001
1	102 (48.8%)	37 (77.1%)	65 (40.4%)	
2	79 (37.8%)	10 (20.8%)	69 (42.9%)	
3	28 (13.4%)	1 (2.1%)	27 (16.8%)	
CT pattern				0.062
Probable UIP	34 (16.3%)	12 (25%)	22 (13.7%)	
Definite UIP	175 (83.7%)	36 (75%)	139 (86.3%)	
Blood pressure				
SBP (mmHg)	128 ± 20	126 ± 20	128 ± 18	0.615
DBP (mmHg)	78 (72–84)	83 (72–87)	77 (72–82)	0.033
Treatment				
Treatment of CVD				0.753
No	52 (24.9%)	6 (12.5%)	46 (28.6%)	
Yes	157 (75.1%)	42 (87.5%)	115 (71.4%)	
Anti-fibrotic treatment				0.753
No	126 (60.3%)	28 (58.3%)	98 (60.9%)	
Yes	83 (39.7%)	20 (41.7%)	63 (39.1%)	
Anti-acid therapies				<0.001
No	146 (69.9%)	43 (89.6%)	103 (64%)	
Yes	63 (30.1%)	5 (10.4%)	58 (36%)	
Long-term home oxygen treatment				0.973
No	118 (56.5%)	27 (56.3%)	91 (56.5%)	
Yes	91 (43.5%)	21 (43.8%)	70 (43.5%)	
Hospital stay duration (days)	8 (6–10)	7 (5–9)	8 (6–10)	0.025

Data are presented as the mean ± SD, *n* (%) or median (IQR). Student’s *t*-test, Pearson’s chi-square test and Mann–Whitney U test were used to compare differences between groups. BMI, body mass index; CVD, cardiovascular disease; COPD, chronic obstructive pulmonary disease‌; ALB, albumin; CRP, c-reactive protein; PNI, prognostic nutritional index; CAR, CRP to ALB ratio; LCR, lymphocyte to CRP ratio; FVC, forced vital capacity; FEV1, forced expiratory volume in the 1st second; DLCO, diffusion capacity for carbon monoxide; GAP, gender-age-physiology stages; CT, computed tomography; UIP, usual interstitial pneumonia; SBP, systolic blood pressure; DBP, diastolic blood pressure.

### Correlation of PNI, CAR, and LCR with pulmonary function, age, and BMI

The decline in pulmonary function, advancing age, and low body mass index (BMI) are known to adversely impact the prognosis of IPF [17–20]. Therefore, we assessed the correlations (Spearman’s rho and *p*-values) of PNI, CAR and LCR with these parameters, as presented in Table 2. Our findings indicate that PNI is positively correlated with DLCO% of predicted and BMI, while it is negatively correlated with age and shows no significant correlation with FVC% of predicted. Conversely, CAR is positively correlated with age, negatively correlated with both DLCO% of predicted and FVC% of predicted, and exhibits no association with BMI. LCR, on the other hand, is positively correlated with FVC% of predicted, DLCO% of predicted, and BMI, while negatively correlated with age.

**Table 2.** Correlations of PNI, CAR and LCR with pulmonary function, age and BMI.

	FVC% pre	DLCO% pre	Age	BMI
PNI	Rho = 0.121	Rho = 0.196	Rho = −0.29	Rho = 0.22
	*p* = 0.061	*p* = 0.002	*p* < 0.001	*p* < 0.001
CAR	Rho = −0.128	Rho = −0.256	Rho = 0.21	Rho = −0.12
	*p* = 0.047	*p* < 0.001	*p* = 0.001	*p* = 0.074
LCR	Rho = 0.136	Rho = 0.288	Rho = −0.24	Rho = 0.13
	*p* = 0.035	*p* < 0.001	*p* < 0.001	*p* = 0.042

BMI, body mass index; PNI, prognostic nutritional index; CAR, CRP to ALB ratio; LCR, lymphocyte to CRP ratio.

### Diagnostic accuracy of PNI, CAR and LCR

**Figure 2.**
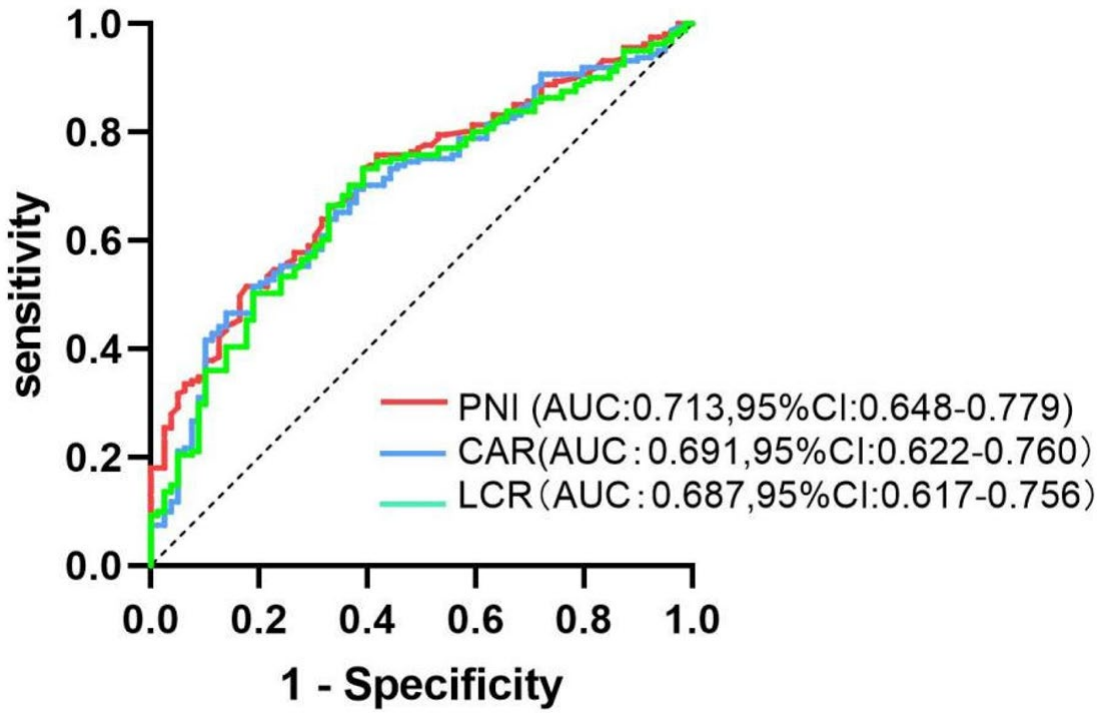
ROC curves of PNI, CAR and LCR in predicting overall mortality (non-survival) in IPF patients. PNI, prognostic nutritional index; CAR, CRP to ALB ratio; LCR, lymphocyte to CRP ratio.

**Figure 3.**
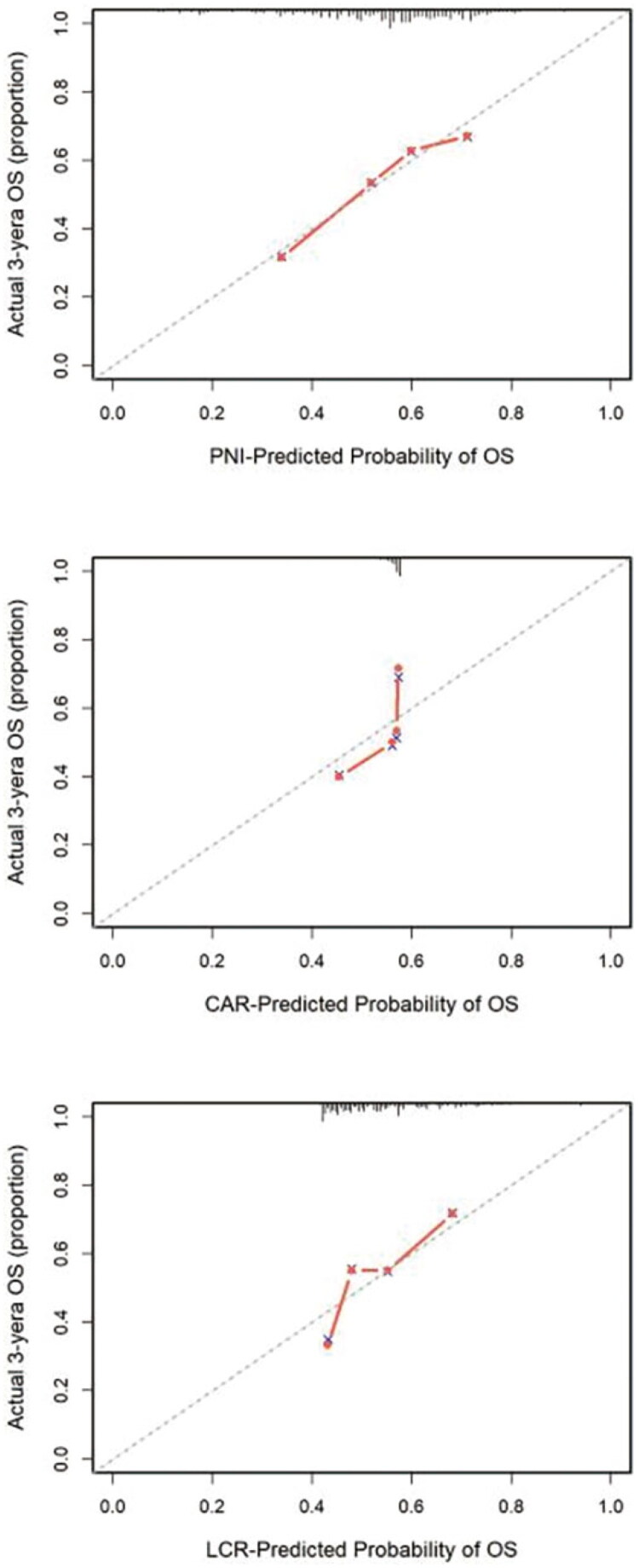
3-Year calibration curves of PNI, CAR and LCR in IPF patients. PNI, prognostic nutritional index; CAR, CRP to ALB ratio; LCR, lymphocyte to CRP ratio.

For overall mortality (OS), ROC curves showed that PNI had the highest AUC (0.715, 95% CI: 0.648–0.779) among the three indices, followed by CAR (0.691, 95% CI: 0.622–0.760) and LCR (0.687, 95% CI: 0.617–0.756) (Figure 2). Additionally, the 3-year calibration curve results indicated that PNI provided superior survival predictions for OS in IPF patients compared to CAR and LCR (Figure 3).

### Survival outcomes according to PNI, CAR and LCR

**Figure 4.**
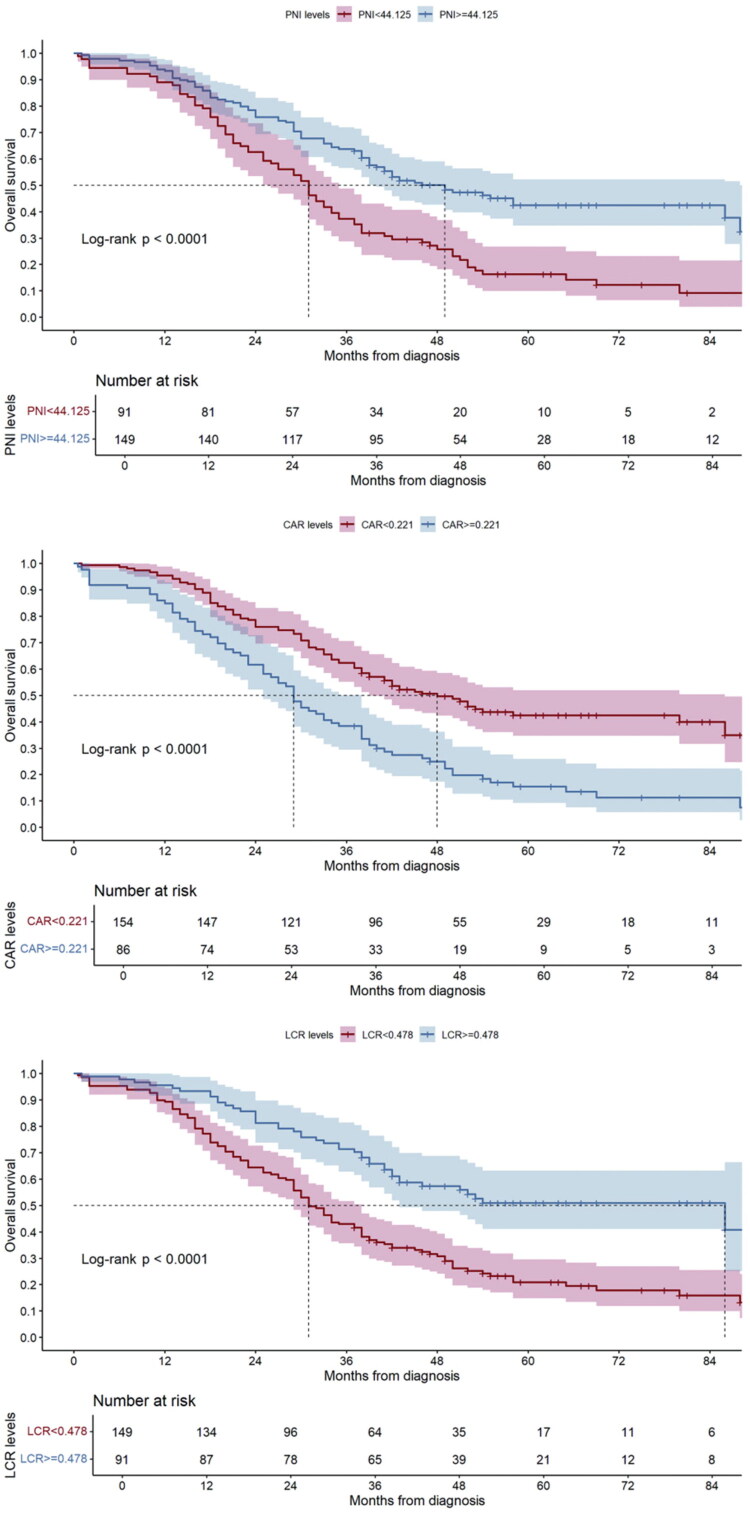
Kaplan–Meier curves for overall survival time by PNI, CAR and LCR. PNI, prognostic nutritional index; CAR, CRP to ALB ratio; LCR, lymphocyte to CRP ratio.

Based on the ROC curves, the cut-off values for PNI, CAR and LCR were 44.125, 0.221 and 0.478 respectively. These indices were subsequently transformed into binary variables, allowing for subgroup analysis based on these cut-off values. The Kaplan-Meier survival curves revealed that the high PNI group (PNI > 44.125), high LCR group (LCR > 0.786), and low CAR group (CAR < 0.221) had significantly higher survival rates (*p* < 0.0001) (Figure 4).

Univariate Cox regression analyses indicated that high PNI, low CAR, and high LCR were significantly associated with longer OS. After adjusting for confounding factors, including age (≥65 years), BMI (≥24), neutrophil count, FVC% of predicted and DLCO% of predicted, high PNI was associated with a 47% decrease in mortality risk (Model 2: *p* = 0.020, HR = 1.470, 95% CI: 1.063–2.032). However, CAR and LCR did not show significant associations with mortality risk in Model 2 (Table 3).

**Table 3.** Univariate and multivariable cox regression analysis of PNI, CAR and LCR for non-survivors as the outcome in IPF patients.

	Model 1			Model 2		
Variables	HR	95% CI	*p* Value	HR	95% CI	*p* Value
By PNI cut-off						
PNI < 44.125	2.019	1.479–2.756	<0.001	1.470	1.063–2.032	0.020
By CAR cut-off						
CAR < 0.221	0.461	0.338–0.629	<0.001	0.767	0.535–1.098	0.147
By LCR cut-off						
LCR < 0.478	2.341	1.648–3.325	<0.001	1.332	0.907–1.955	0.143

HR: hazard ratio; 95% CI: 95% confidence interval. Model 1: unadjusted. Model 2: adjusted for age, BMI, neutrophil count, FVC% of predicted and DLCO% of predicted. PNI, prognostic nutritional index; CAR, CRP to ALB ratio; LCR, lymphocyte to CRP ratio.

### Differential exploration of PNI, CAR, and LCR in the survivors and IPF-related deaths

The clinical features of the 48 survivors and 113 IPF-related deaths are detailed in Table 4. Notably, PNI, CAR and LCR were markedly different between the survivors and IPF-related deaths (*p* < 0.001). Furthermore, distinct differences in computed tomography (CT) patterns were seen in survivors and IPF-related deaths compared to survivors and non-survivors, while no significant differences were observed in the treatment of CVD and SBP.

**Table 4.** Comparison of clinical features of survivors and IPF-related deaths in IPF patients.

Variables	Total (*n* = 161)	Survivors (*n* = 48)	IPF-related death (*n* = 113)	*p*
Demographics				
Age	64.6 ± 9.8	58.3 ± 9.3	67.2 ± 8.8	<0.001
Gender				0.733
Male	117 (72.7%)	34 (70.8%)	83 (73.5%)	
Female	44 (27.3%)	14 (29.2%)	30 (26.5%)	
Smoke status				0.758
Current smoking	10 (6.2%)	2 (4.2%)	8 (7.1%)	
Former smoking	97 (60.2%)	30 (62.5%)	67 (59.3%)	
Never smoke	54 (33.5%)	16 (33.3%)	38 (33.6%)	
BMI, kg/m^2^	23.64 ± 3.61	25.54 ± 3.12	22.83 ± 3.52	<0.001
Comorbidity				
CVD, *n* (%)				0.048
No	117 (72.7%)	40 (83.3%)	77 (68.1%)	
Yes	44 (27.3%)	8 (16.7%)	36 (31.9%)	
Diabetes, *n* (%)				0.434
No	139 (86.3%)	43 (89.6%)	96 (85%)	
Yes	22 (13.7%)	5 (10.4%)	17 (15%)	
Pulmonary emphysema, *n* (%)				0.723
No	142 (88.2%)	43 (89.6%)	99 (87.6%)	
Yes	19 (11.8%)	5 (10.4%)	14 (12.4%)	
COPD, *n* (%)				0.306
No	147 (91.3%)	46 (95.8%)	101 (89.4%)	
Yes	14 (8.7%)	2 (4.2%)	12 (10.6%)	
Tuberculosis, *n* (%)				0.078
No	146 (90.7%)	47 (97.9%)	99 (87.6%)	
Yes	15 (9.3%)	1 (2.1%)	14 (12.4%)	
Lung infection				0.101
No	128 (79.5%)	42 (87.5%)	86 (76.1%)	
Yes	33 (20.5%)	6 (12.5%)	27 (23.9%)	
Respiratory failure				0.002
No	141 (87.6%)	48 (100%)	93 (82.3%)	
Yes	20 (12.4%)	0 (0%)	20 (17.7%)	
Laboratory results				
Hemoglobin (g/L)	131.2 ± 17.5	138.3 ± 16.3	129.6 ± 17.4	0.004
Lymphocyte (×109 /L)	1.77 ± 0.65	1.93 ± 0.54	1.70 ± 0.68	0.047
Neutrophil (×109 /L)	4.49 (3.45–6.24)	4.25 (3.66–5.52)	4.69 (3.38–6.49)	0.258
Platelet (×10*9/L)	203.0 (164.5–245.5)	211.0 (172.3–240.0)	202.0 (158.0–247.5)	0.461
ALB (g/L)	36.30 (33.45–38.50)	38.20 (35.83–39.78)	35.60 (32.25–37.35)	<0.001
CRP (mg/L)	4.710 (2.805–10.650)	3.235 (2.235–5.393)	5.800 (3.225–15.650)	<0.001
Laboratory results-derived indexes				
PNI	44.445 ± 5.834	47.468 ± 3.985	43.162 ± 6.031	<0.001
CAR	0.131 (0.074–0.360)	0.089 (0.059–0.146)	0.166 (0.092–0.465)	<0.001
LCR	0.393 (0.131–0.664)	0.574 (0.330–0.870)	0.281 (0.094–0.549)	<0.001
Pulmonary function tests				
FVC% of predicted	80.6 ± 21.8	89.1 ± 18.4	76.9 ± 22.2	<0.001
FEV1% of predicted	83.2 ± 20.5	89.9 ± 19.8	80.3 ± 20.1	0.006
DLCO% of predicted	53.0 (40.0–65.8)	63.2 (50.6–79.3)	50 (40.0–59.5)	<0.001
FEV1/FVC	0.833 (0.780–0.886)	0.819 (0.771–0.855)	0.839 (0.786–0.907)	0.102
GAP index				<0.001
1	82 (51.2%)	37 (77.1%)	45 (40.2%)	
2	58 (36.3%)	10 (20.8%)	48 (42.9%)	
3	20 (12.5%)	1 (2.1%)	19 (17.0%)	
CT pattern				0.019
Probable UIP	24 (14.9%)	12 (25%)	12 (10.6%)	
Definite UIP	137 (85.1%)	36 (75%)	101 (89.4%)	
Blood pressure				
SBP (mmHg)	123 (112–137)	120 (113–137)	123 (112–137)	0.772
DBP (mmHg)	79 ± 11	81 ± 11	78 ± 11	0.068
Treatment				
Treatment of CVD				0.412
No	135 (83.9%)	42 (87.5%)	93 (82.3%)	
Yes	157 (75.1%)	42 (87.5%)	115 (71.4%)	
Anti-fibrotic treatment				0.232
No	105 (65.2%)	28 (58.3%)	77 (68.1%)	
Yes	56 (34.8%)	20 (41.7%)	36 (31.9%)	
Anti-acid therapies				0.008
No	122 (75.8%)	43 (89.6%)	79 (69.9%)	
Yes	39 (24.2%)	5 (10.4%)	34 (30.1%)	
Long-term home oxygen treatment				0.954
No	90 (55.9%)	27 (56.3%)	63 (55.8%)	
Yes	71 (44.1%)	21 (43.8%)	50 (44.2%)	
Hospital stay duration (days)	8 (6–10)	7 (5–8)	8 (6–10)	0.024

Data are presented as the mean ± SD, *n* (%) or median (IQR). Student’s *t*-test, Pearson’s chi-square test and Mann–Whitney U test were used to compare differences between groups. BMI, body mass index; CVD, cardiovascular disease; COPD, chronic obstructive pulmonary disease‌; ALB, albumin; CRP, c-reactive protein; PNI, prognostic nutritional index; CAR, CRP to ALB ratio; LCR, lymphocyte to CRP ratio; FVC, forced vital capacity; FEV1, forced expiratory volume in the 1st second; DLCO, diffusion capacity for carbon monoxide; GAP, gender-age-physiology stages; CT, computed tomography; UIP, usual interstitial pneumonia; SBP, systolic blood pressure; DBP, diastolic blood pressure.

**Figure 5.**
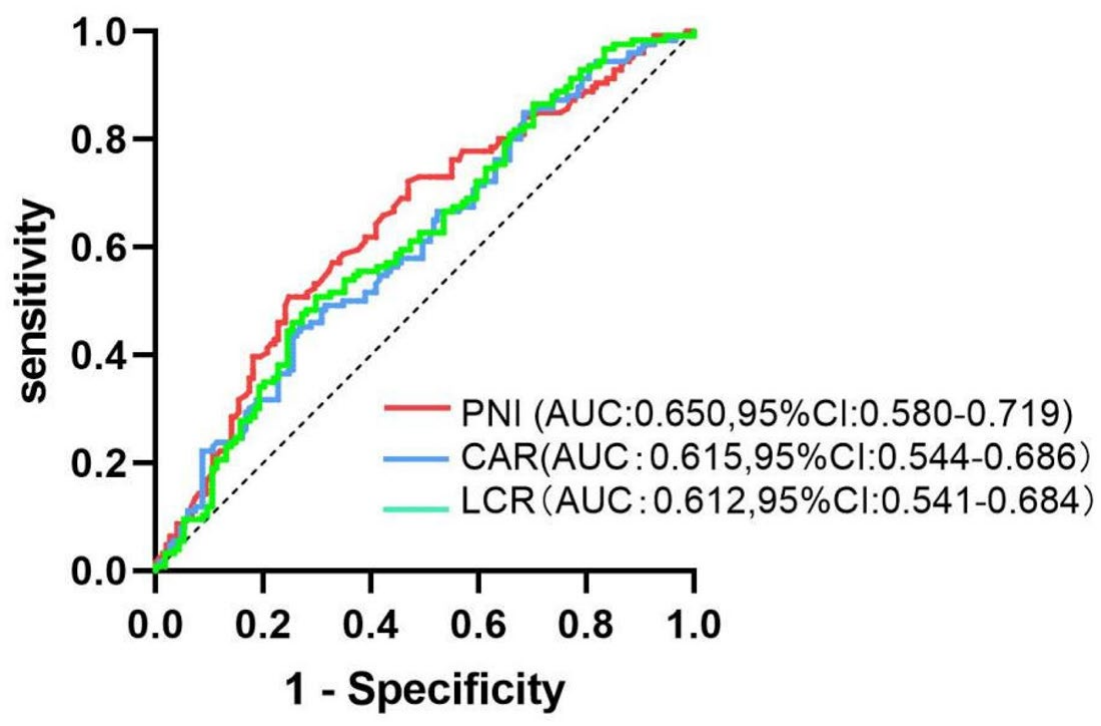
ROC curves of PNI, CAR and LCR in predicting the IPF-related mortality in IPF patients. PNI, prognostic nutritional index; CAR, CRP to ALB ratio; LCR, lymphocyte to CRP ratio.

In the ROC analysis, PNI demonstrated predictive capacity for IPF-related mortality, achieving an AUC of 0.650 (95% CI, 0.580–0.719, *p* < 0.001). Similarly, CAR predicted IPF-related mortality with an AUC of 0.615 (95% CI: 0.544–0.686, *p* = 0.002), while LCR exhibited an AUC of 0.612 (95% CI: 0.541–0.684, *p* = 0.003) (Figure 5).

From the analysis presented in Table 5, both PNI and LCR were identified as protective factors against IPF-related mortality in univariate Cox regression analysis, whereas CAR emerged as a risk factor. After adjusting for confounding variables, including age (≥65 years), BMI (≥24), neutrophil count, FVC% of predicted and DLCO% of predicted, it was determined that for every 1.00 increase in PNI, the risk of IPF-related mortality decreased by 4.5% (Model 2: *p* = 0.015, HR = 0.955, 95% CI: 0.921–0.991). However, CAR and LCR did not show significant associations with the risk of IPF-related mortality in Model 2.

**Table 5.** Univariate and multivariable cox regression analysis of PNI, CAR and LCR for IPF-related deaths as the outcome in IPF patients.

	Model 1			Model 2		
Variables	HR	95% CI	*p* Value	HR	95% CI	*p* Value
PNI	0.916	0.886-0.947	<0.001	0.955	0.921–0.991	0.015
CAR	1.297	1.144-1.469	<0.001	1.130	0.954-1.339	0.157
LCR	0.491	0.297-0.810	0.005	0.908	0.582-1.418	0.672

HR: hazard ratio; 95% CI: 95% confidence interval. Model 1: unadjusted. Model 2: adjusted for age, BMI, neutrophil count, FVC% of predicted and DLCO% of predicted. PNI, prognostic nutritional index; CAR, CRP to ALB ratio; LCR, lymphocyte to CRP ratio.

## Discussion

The present study evaluated the prognostic value of PNI, CAR, and LCR in IPF patients. Key findings include: (1) Non-survivors and IPF-related deaths exhibited significantly lower PNI and LCR, and higher CAR compared to survivors; (2) PNI showed the strongest correlation with age and BMI, while LCR was most strongly associated with pulmonary function; (3) ROC and survival analyses demonstrated that PNI had superior predictive accuracy for OS and IPF-related mortality compared to CAR and LCR; (4) Multivariable Cox regression confirmed that PNI, but not CAR or LCR, was an independent predictor of both overall survival and IPF-related mortality after adjusting for confounding factors. IPF is a chronic, progressive lung disease predominantly affecting older males, characterized by a poor prognosis and frequent exacerbation of dyspnea, leading to persistent decline in lung function and ultimately death [21,22]. In our cohort, up to 74.2% of IPF patients were male, with a median age at diagnosis of 67 years and a mean follow-up period of 29 months for non-survivors. The pathogenesis of IPF is multifaceted, characterized by persistent inflammation within the respiratory tract and immune dysregulation, both of which significantly contribute to disease progression [23,24]. Lymphocytes, as a pivotal component of the immune system, are notably affected by aging, leading to diminished lymphocyte proliferation and adaptive immune responses, alongside an increase in inflammatory processes [25]. Moreover, the immune system plays a crucial role in modulating the onset and resolution of inflammatory responses, further influencing the development of inflammation [26]. CRP serves as a classic clinical parameter reflecting the severity of inflammation and has recently been recognized for its role as a regulatory mediator in autoimmune responses [27,28]. ALB, a key indicator of nutritional status, has been associated with poor prognostic outcomes in older adults when serum levels are low [29]. Consequently, composite indices such as the PNI, CAR and LCR are derived from the interplay of CRP, lymphocytes, and ALB. These indices may provide valuable insights into the intricate relationships among inflammation, immunity, and nutrition in the context of IPF.

PNI has demonstrated utility in predicting outcomes in lung cancer [30]. In this study, we observed significantly higher PNI levels in survivors compared to non-survivors and IPF-related deaths. Furthermore, elevated PNI was associated with favorable prognoses in IPF patients, corroborating previous findings in connective tissue disease-related interstitial lung diseases (CTD-ILD) [31]. CAR is increasingly recognized for its prognostic value in lung diseases, including pneumonia, lung cancer, chronic obstructive pulmonary disease (COPD), and idiopathic inflammatory myopathy-associated interstitial lung disease (IIM-ILD). Consistent with our findings, elevated CAR frequently indicates poor prognosis [14,15,32,33]. LCR, an emerging biomarker that reflects the interplay of inflammation, immunity, and nutrition, has been associated with postoperative complications and infectious diseases across various malignancies; however, its role in IPF remains underexplored [16,34]. Our results indicate that higher LCR correlates with improved outcomes, aligning with prior studies. Notably, baseline analysis revealed that non-survivors and IPF-related deaths had extended hospital stays. Prolonged hospitalization (>7 days) is linked to increased mortality in CTD-ILD [35]. Furthermore, a study by Joshua J et al. indicated that aging correlates with shorter hospital stays in IPF patients [36]. And our findings showed that non-survivors were significantly older, potentially due to comorbid cardiopulmonary diseases and exacerbations in hospitalized patients. Another consideration is that there was no significant difference in antifibrotic therapy between survivors and non-survivors. Notably, while pirfenidone and nintedanib received FDA approval for IPF treatment in October 2014, the study included patients from January 2011 to December 2020 – a period marked by insufficient disease awareness, coupled with the drugs’ high cost (as they were not covered by medical insurance at the time). Given that IPF is a chronic disease requiring lifelong medication, such financial burdens, compounded by frequent acute exacerbations necessitating hospitalization [37,38], likely constrained treatment adherence. This, in turn, would have affected patient compliance to a certain extent. From the collected data, the median duration of antifibrotic treatment was relatively short (14.2 months); furthermore, some patients required dose adjustments due to tolerance issues. These factors may have collectively hindered the manifestation of the drugs’ effects on long-term survival outcomes. Additionally, there was considerable heterogeneity in baseline pulmonary function impairment among the IPF patients in this study (with FVC% predicted ranging from 32% to 89%), suggesting that the survival benefits of the drugs may be more pronounced in specific subgroups, such as those with moderate to severe declines in pulmonary function.

CAR and LCR are overly dependent on CRP, a non-specific inflammatory marker that fluctuates markedly due to infection, acute exacerbation, and comorbidities [5]. In contrast, albumin and lymphocytes are relatively stable in chronic diseases, making PNI less susceptible to short-term disturbances [39]. Meanwhile, malnutrition is highly prevalent in patients with IPF and carries important prognostic significance [40]. Furthermore, the common gastrointestinal adverse reactions of pirfenidone and nintedanib may chronically impair nutrient absorption and exacerbate malnutrition and immune dysfunction [41]. Together, these mechanisms explain why PNI exhibits superior prognostic performance compared with CAR and LCR.

Our investigation into the correlation between PNI, CAR, and LCR with pulmonary function, age, and BMI revealed that PNI was most strongly correlated with age and BMI, while LCR correlated strongly with FVC% and DLCO%. ROC analysis demonstrated that PNI exhibited the highest AUC for predicting overall mortality (0.713) and IPF-related mortality (0.650), outperforming CAR (0.691 and 0.615) and LCR (0.687 and 0.612). Calibration curves further supported the predictive capacity of PNI for 3-year survival in IPF patients. Survival analysis, stratifying PNI, CAR, and LCR into high and low groups based on the cut-off values determined by the ROC curves, indicated that lower PNI and LCR and higher CAR corresponded to poorer survival outcomes. This association may relate to elevated serum CRP levels in IPF patients and decreased immune function linked to reduced lymphocyte counts [42,43]. Additionally, lower serum albumin levels in non-survivors reflect a state of malnutrition, exacerbating disease progression [9]. Furthermore, only PNI remained an independent predictor of OS and IPF-related mortality in multivariate models, whereas CAR and LCR lost significance after adjusting for confounding factors. This suggests that the associations of CAR and LCR with survival may be mediated by other variables (e.g. pulmonary function, age) or confounded by factors not fully accounted for, limiting their utility as independent prognostic markers. In contrast, PNI remained an independent predictor, highlighting its superior robustness as a prognostic indicator in IPF. The attenuation of CAR and LCR’s predictive power in multivariate models likely reflects their collinearity with confounding factors. For instance, CRP – a key component of both CAR and LCR – is tightly linked to systemic inflammation, which in turn correlates with advancing age [44]. In addition, CAR and LCR may be more applicable during the inflammatory response phase in the pathogenesis of IPF, a hypothesis that warrants further investigation [45].

The clinical outcomes of IPF patients exhibit significant variability, underscoring the need for reliable biomarkers to assess prognosis and guide treatment decisions. Compared to pulmonary function tests and high-resolution computed tomography (HRCT), PNI, CAR, and LCR are more readily obtainable, quicker, and cost-effective, particularly in primary care and community hospital settings where advanced diagnostic equipment may be limited.

### Limitations

This study also has some limitations. Firstly, its retrospective design inherently carries the risk of incomplete data and recall bias. Secondly, being a single-center study with a limited sample size, further prospective multicenter studies are warranted to validate the predictive roles of PNI, CAR, and LCR in IPF prognosis. Lastly, we lacked dynamic data on these biomarkers over the follow-up period, primarily relying on telephone follow-ups, which limits our ability to assess the relationship between temporal changes in PNI, CAR, and LCR and disease progression, potentially affecting the generalizability of our findings.

## Conclusion

This study demonstrated that low levels of PNI and LCR, and high levels of CAR were strongly associated with overall mortality and IPF-related mortality in IPF patients. The predictive effect of PNI is stronger than that of CAR and LCR. Our study is informative in providing prognosis and clinical management of IPF patients.

## Data Availability

Data supporting the results of this study are available from the corresponding author upon reasonable request.
